# Programmable Unlocking Nano‐Matryoshka‐CRISPR Precisely Reverses Immunosuppression to Unleash Cascade Amplified Adaptive Immune Response

**DOI:** 10.1002/advs.202100292

**Published:** 2021-05-14

**Authors:** Jin Yang, Zhike Li, Meiling Shen, Yan Wang, Li Wang, Jiamiao Li, Wen Yang, Jie Li, Haijun Li, Xinxin Wang, Qinjie Wu, Changyang Gong

**Affiliations:** ^1^ State Key Laboratory of Biotherapy and Cancer Center West China Hospital Sichuan University Chengdu 610041 P. R. China

**Keywords:** cancer immunotherapy, cascade amplified, CRISPR/Cas9, nano‐matryoshka, programmable unlocking

## Abstract

Immune checkpoint blockade (ICB) is an attractive option in cancer therapy, but its efficacy is still less than expected due to the transient and incomplete blocking and the low responsiveness. Herein, an unprecedented programmable unlocking nano‐matryoshka‐CRISPR system (PUN) targeting programmed cell death ligand 1 (PD‐L1) and protein tyrosine phosphatase N2 (PTPN2) is fabricated for permanent and complete and highly responsive immunotherapy. While PUN is inert at normal physiological conditions, enzyme‐abundant tumor microenvironment and preternatural intracellular oxidative stress sequentially trigger programmable unlocking of PUN to realize a nano‐matryoshka‐like release of CRISPR/Cas9. The successful nucleus localization of CRISPR/Cas9 ensures the highly efficient disruption of PD‐L1 and PTPN2 to unleash cascade amplified adaptive immune response via revoking the immune checkpoint effect. PD‐L1 downregulation in tumor cells not only disrupts PD‐1/PD‐L1 interaction to attenuate the immunosurveillance evasion but also spurs potent immune T cell responses to enhance adaptive immunity. Synchronously, inhibition of JAK/STAT pathway is relieved by deleting PTPN2, which promotes tumor susceptibility to CD8^+^ T cells depending on IFN‐*γ*, thus further amplifying adaptive immune responses. Combining these advances together, PUN exhibits optimal antitumor efficiency and long‐term immune memory with negligible toxicity, which provides a promising alternative to current ICB therapy.

## Graphical abstract

Programmable unlocking nano‐matryoshka‐CRISPR (PUN) possesses multistage sensitive properties and exhibits excellent performances, including prolonged blood circulation, precise tumor recognition, deep tumor penetration, robust lysosomal escape, and effective transfection. With the precise control of CRISPR/Cas9 activation, PUN realizes complete and thorough intracellular disruption of PD‐L1 and PTPN2, thus eliciting cascade amplified adaptive immune response to boost antitumor immune effects.

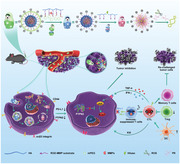

## 1 Introduction

Immune checkpoint blockade (ICB) has shown potential clinical advantages in cancer therapy by evoking the immune system.^[^ ^1^ ^]^ Conventional approach of ICB is focused on monoclonal antibody‐based therapy, such as antibodies against programmed cell death‐1(PD‐1), programmed cell death ligand‐1 (PD‐L1), cytotoxic T lymphocyte‐associated antigen‐4 (CTLA4), etc. ^[^ ^2^ ^]^ Unfortunately, the transient duration and instability of antibodies after systemic administration, together with the severe “on‐target but off‐tumor” problem, lead to a low response rate and overshadow their application prospects.^[^ ^3^ ^]^ RNA interference provides an alternative approach to disturb checkpoints interaction, but it still encounters the problem of transient and incomplete effectiveness.^[^ ^4^ ^]^ Thus, it would be of great interests to explore a more permanent, complete and highly responsive therapeutic strategy.

The clustered regularly interspaced short palindromic repeat (CRISPR)/CRISPR‐associated protein 9 (CRISPR/Cas9) system has emerged as a cutting‐edge genome editing tool, which can permanently attenuate the expression of target genes under the guidance of sgRNA and Cas9.^[^ ^5^ ^]^ Notably, different from immune checkpoint inhibitors and RNA therapy, CRISPR/Cas9 edited tumor cells and their progenies will lose the original copy of oncogenes, thus being vulnerable to attack by the immune system. ^[^ ^6^ ^]^ This suggests that CRISPR/Cas9‐based blockade could serve as a permanent and thoroughly strategy to evoke more effective and durable antitumor immunity by knocking down genes targeting immune checkpoints.

PD‐L1 has been identified as a key regulator of immune evasion that ultimately leads to immune tolerance and further deterioration of tumor.^[^ ^7^ ^]^ Accumulating evidences have suggested that CRISPR/Cas9 regulating expression of PD‐L1 can contribute to activation of adaptive immunity, but the outcome of monotherapy targeted to PD‐L1 would be compromised by the low immune response of tumor cells. ^[^ ^8^ ^]^ Taking this into account, much effort has been devoted to combinative blockade of multiple immune checkpoints. Overexpressed protein tyrosine phosphatase N2 (PTPN2) renders tumor resistant to immunotherapy by negatively regulating IFN‐*γ* signaling with JAK/STAT pathway.^[^ ^9^ ^]^ On the one hand, downregulating PD‐L1‐positive cells via CRISPR/Cas9 can revive the slumbering T cells and elicit T cell‐mediated adaptive immunity.^[^ ^10^ ^]^ On the other hand, deficiency of PTPN2 can improve recognition of tumor cells and susceptibility to cytotoxic CD8^+^ T cells by invoking an IFN‐*γ* response. ^[^ ^11^ ^]^ Thus, we speculated that combinative blockade of PD‐L1 and PTPN2 in tumor cells using CRISPR/Cas9 could reverse immunosuppression to unleash cascade amplified adaptive immune response. However, PD‐L1 and PTPN2 are not only overexpressed in tumor cells but also exist in other immune cells and tissue cells, which may give rise to undesirable safety issues after systemic administration.^[^ ^12^ ^]^ Besides, as a targeted nuclease editing toolbox, various delivery obstacles before CRISPR/Cas9 reaching the destination make the dilemma even worse.^[^ ^13^ ^]^ Therefore, the development of feasible strategy to specifically and efficiently delivery CRISPR/Cas9 to target sites for activation of adaptive immunity has become a priority.

**Scheme 1:**
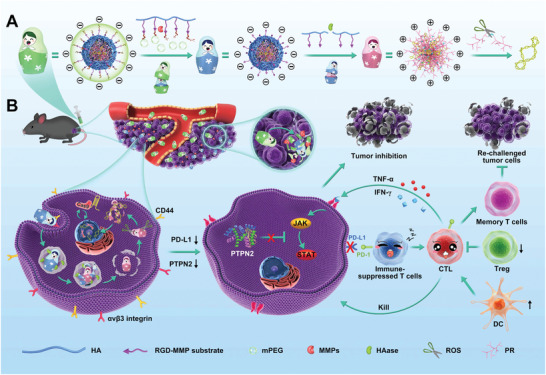
Design and immunotherapeutic functions of PUN@Cas‐PT. A) Fabrication and the programmable unlocking process of PUN@Cas‐PT in response to MMPs, HAase, and ROS. B) Schematic illustration of the utilization of PUN@Cas‐PT for efficient multitargeted ICB therapy in vivo.

Herein, a programmable unlocking nano‐matryoshka‐CRISPR system targeting PD‐L1 and PTPN2 (PUN@Cas‐PT) was designed based on the characterizations of the tumor microenvironment (TME) for permanent, complete, and highly responsive immunotherapy, which own hierarchical responsive property for precise and efficient control of CRISPR/Cas9 activation (**Scheme** **1**). PUN@Cas‐PT consisted of multi‐enzyme‐responsive corona and oxidative stress‐sensitive core. The design of matryoshka‐like structure endows PUN@Cas‐PT with stealth property in circulation, enhanced tumor retention and internalization, as well as enables a charge reversion for effective endosome escape and rapid release of CRISPR/Cas9 for synchronous, permanent, and complete multi‐genes disruption. Like a programmable unlocking “nano‐matryoshka” way, PUN@Cas‐PT can overcome sequential biological barriers and only release the CRISPR/Cas9 in tumor sites which are characterized by overexpressed metalloproteases (MMPs), hyaluronidase (HAase), and high endogenous reactive oxygen species (ROS) concentration. ^[^ ^14^ ^]^ As for the multistage responsiveness, the negatively charged PUN@Cas‐PT is inert in prolonged blood circulation owing to PEGylation, while achieving the first unlocking process at the MMPs‐rich tumor microenvironment to exert enhanced tumor recognition, deep penetration, and cellular internalization depending on exposed RGD and HA. The second unlocking is completed in lyso/endosomes, in which HAase triggers a charge reversion by degrading HA to facilitate rapid lysosomal escape. High endogenous ROS turns on the last unlocking for effective release of payload. The released CRISPR/Cas9 can rapidly locate in nucleus and simultaneously downregulated PD‐L1 and PTPN2 to unleash cascade amplified adaptive immune response via revoking the immune checkpoint effect. To validate this hypothesis, the antitumor efficacy and safety of PUN@Cas‐PT were investigated both in vitro and in vivo. This work presented here provides a new weapon against cancer, which holds great promises for advanced application in the field of antitumor immunotherapy.

## 2 Results and Discussion

To prepare matryoshka‐like PUN, ROS‐sensitive polyethyleneimine (PEI) derivative (PR) was first synthesized by crosslinking PEI 1.8K with the ROS‐cleavable thioketal linker. The obtained PR showed related characteristic peaks (O=C—NH, 3.3 ppm) in ^1^H nuclear magnetic resonance (^1^H NMR) and peak at 1653 cm^−1^ in Fourier transform infrared (FTIR) spectroscopy (Figure S1, Supporting Information), confirming the success of amidation reaction. Second, the dual‐enzyme‐responsive copolymer hyaluronic acid (HA)‐RGD peptide (Arg‐Gly‐Asp)‐MMPs substrate (GPLGVRG)‐polyethylene glycol (PEG) (HRMP) was produced via a two‐step reaction. To introduce the MMPs cleavable fusion peptide between HA and the long‐chain PEG, the primary amine of peptide was first reacted with the active carboxyl group of HA to form an amide bond. Then, the sulfhydryl group at the other end of the peptide efficiently reacted with methoxy poly(ethylene glycol) maleimide (MPEG‐mal). Characterization by ^1^H NMR and FTIR showed successful formation of the conjugate (Figure S2, Supporting Information).

**Figure 1:**
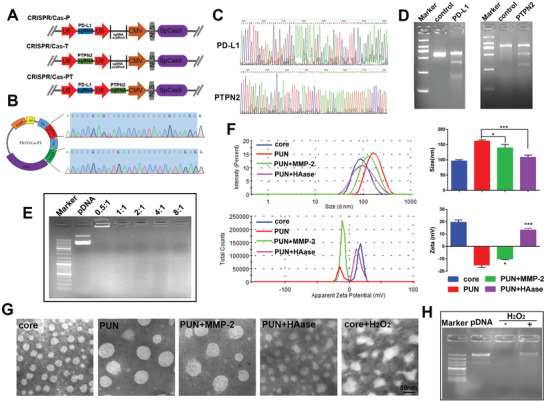
Preparation and characterization of PUN. A) Schematic structure of PX333 vector after inserting sgRNA. B) Sequencing result of the sgRNA targeting PD‐L1 and PTPN2 in CRISPR/Cas‐PT plasmid. C) Sanger sequencing result and D) T7EI cleavage assay of the PCR amplicon of PD‐L1/PTPN2 loci after transfection with the optimal CRIPSR/Cas‐PT. E) 1% agarose gel image of PR/pDNA. Lane 1, DNA ladder; lane 2, naked pDNA, lane 3–7, PR/pDNA at mass ratio of 0.5:1, 1:1, 2:1, 4:1, and 8:1, respectively. F) Hydrodynamic diameter and zeta potential of core, PUN, MMP‐2‐treated PUN (PUN+MMP‐2), and HAase‐treated PUN (PUN+HAase). G) Morphologies of nanoparticles measured by TEM. H) Gel electrophoresis assay of the core before and after incubation in a solution containing 25 × 10^−3^ m H_2_O_2_ and 1.6 × 10^−6^ m CuCl_2_ (*P < 0.05, **P < 0.01, ***P < 0.001).

Next, multitargeting CRISPR/Cas9 system was constructed for editing PD‐L1 and PTPN2. For optimization, sgRNAs targeting PD‐L1 and PTPN2 were designed and separately inserted into PX333 vectors to obtained CRISPR/Cas‐P and CRISPR/Cas‐T (**Figure** **1**A; Figure S3A, Supporting Information). Based on genome sequencing and T7 endonuclease I (T7EI) assay, CRISPR/Cas‐P containing sgRNA2 and CRISPR/Cas‐T containing sgRNA1 were selected as the optimal system with relatively higher indel rates of 20.9% and 27.0%, respectively (Figure S3B,C, Supporting Information). Next, the two optimal sgRNAs were successively integrated into vector (CRISPR/Cas‐PT) and identified through insertion analysis (Figure 1B). The analysis of the genomic DNA suggested that CRISPR/Cas‐PT could lead to noteworthy mutation at both genome loci (Figure 1C). The T7EI image more intuitively confirmed its satisfactory gene editing capability, with indel rate of 21.6% at PD‐L1 locus and 23.3% at PTPN2 locus, which was barely affected by the integration of two sgRNAs (Figure 1D).

Protection of pDNA from degradation by nucleases is the key to ensure effective transfection.^[^ ^15^ ^]^ The PR was capable of excellent pDNA condense ability as evidenced by the gel retardation assay (Figure 1E). In view of the protection of pDNA and appropriate transfection efficiency, PR efficiently complexed with pDNA at the ratio of 4:1 (PR/pDNA, defined as core, w/w) via electrostatic interaction. According to the dynamic light scattering (DLS) analysis (Figure 1F), core could be well‐dispersed in solution with hydrodynamic diameter of 96.5 ± 3.9 nm (PDI 0.31 ± 0.03) and corresponding zeta potential of +19.5 ± 1.9 mV. For PUN, upon surface coated with HRMP at the optimal mass ratio, the particle size increased (161.6 ± 3.9 nm) (PDI 0.17 ± 0.01) and zeta potential sharply shifted to negative (−15.1 ± 1.8 mV), but still presented spherical morphology under transmission electron microscopic (TEM) (Figure 1G).

It appeared that incubation of PUN with MMP‐2, a slight change of particle size occurring from 161.6 ± 3.9 to 139.4 ± 11.2 nm (PDI 0.16 ± 0.06), while the zeta potential remained negative (−10.9 ± 0.5 mV), which suggested the MMP‐2 responsive deshielding of PEG layer (Figure 1F). Moreover, adding HAase to preform PUN turned zeta potential into positive (+13.3 ± 1.3 mV) and reduced the average size significantly (108.6 ± 6.9 nm) (PDI 0.46 ± 0.11) (*P* < 0.001), which attributed to the degradation and shedding of HA layer in response to HAase. The morphological change under TEM was also an evidence of the dual‐enzyme responsiveness and in good agreement with the DLS analysis (Figure 1G). It has been demonstrated that B16‐F10 cells could spontaneously produce intracellular ROS (Figure S4, Supporting Information). As vividly represented by TEM images, the structures of core were disintegrated in the presence of H_2_O_2_ (25 × 10^−3^ m), followed by effective release of the CRISPR/Cas9 plasmid (Figure 1H), which indicated the good ROS responsive ability. These results suggested that PUN had agile enzyme and ROS responsiveness in the simulated extra/intracellular microenvironment of tumor.

**Figure 2:**
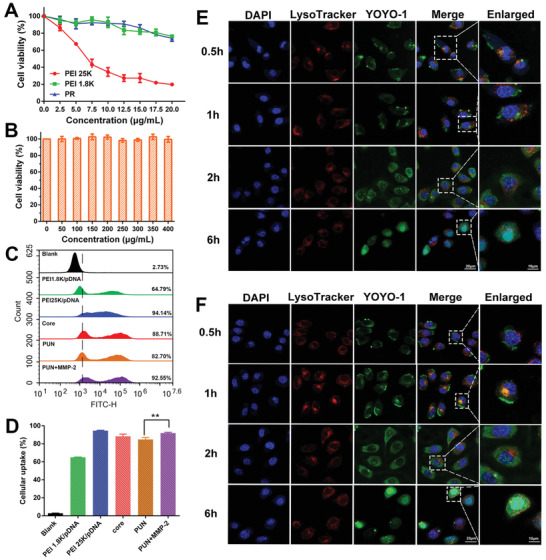
Evaluation of cytotoxicity, cellular uptake, and endosome escape ability. The cellular viability of B16‐F10 cells after being treated by A) PEI 25K, PEI 1.8K, PR and B) HRMP polymer. C) The cellular uptake analysis of PEI 1.8K/pDNA, PEI 25K/pDNA, core, PUN, and PUN+MMP‐2 using flow cytometry and D) quantitative analysis of the corresponding uptake efficiency. Confocal microscope images of B16‐F10 cells after incubating with E) core and F) PUN for 0.5, 1, 2, and 6 h. YOYO‐1 labeled pDNA in green, Lysotracker labeled lyso/endosomes in red, and DAPI labeled nuclei in blue (*P < 0.05, **P < 0.01, ***P < 0.001).

Cytotoxicity is an important concern in the development of gene delivery system.^[^ ^16^ ^]^ MTT assays showed that PR and HRMP had low toxicity, which was significantly lower than PEI 25K (**Figure** **2**A,B; Figure S5, Supporting Information). Owing to the low toxic components, PUN did not show obvious hemolysis at various mass ratios (<5%), which indicates PUN had a good blood compatibility and could serve as a safe system (Figure S6, Supporting Information).

Although PEGylation has the potential to improve the in vivo usability of nanocomplex, it can also hamper their internalization.^[^ ^17^ ^]^ To evaluate the uptake efficiency of PUN, intracellular fluorescence in B16‐F10 cells after 1 h incubation was analyzed by flow cytometry (Figure 2C,D). It has been demonstrated that the expression of CD44 on B16‐F10 cells was nearly 100% (Figure S7, Supporting Information). Benefiting from the interaction between HA backbone of PUN and abundant CD44 receptors on B16‐F10 cells, PUN exhibited a high intracellular uptake efficiency (≈82%). Moreover, MMP‐2 pretreated PUN exhibited much higher internalization efficiency (≈93%) (*P* < 0.01), which may attribute to the fact that MMPs‐sensitive deshielding of PEG would re‐expose RGD peptide and thereby enhance uptake of PUN by binding with the integrin *α*v*β*3 receptors and neuropilin‐1 on B16‐F10 cells.

After endocytosis, effective lyso/endosomal escape plays an important role in gene expression.^[^ ^18^ ^]^ The confocal laser scanning microscope (CLSM) images revealed that core rapidly localized at lyso/endosome and triggered endosome escape within 1 h owing to its “proton sponge” effect (Figure 2E). In comparison, PUN showed delayed colocalization with lyso/endosome (yellow dots) at 1 h because of the shielding effect of PEG layer. A sharp decrease of yellow dots at 2 h indicated that PUN gradually separated from lyso/endosome and released in cytoplasm. Eventually, YOYO‐1 labeled pDNA in PUN accumulated into the nucleus at 6 h (Figure 2F), suggesting the satisfactory lyso/endosome escape and nuclear internalization capability of PUN.

**Figure 3:**
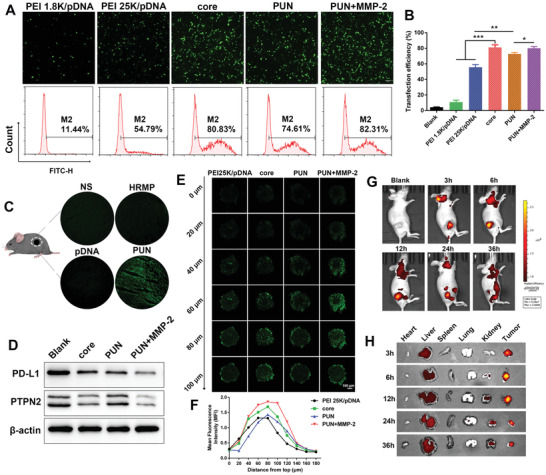
Gene editing efficiency and biodistribution. A) The fluorescence images and B) quantitative analysis of B16‐F10 cells after transfection with PEI 1.8K/pDNA, PEI 25K/pDNA, core, PUN, and PUN+MMP‐2. C) Confocal images of EGFP‐positive cells in tumor slices from mice in NS, HRMP, pDNA, and PUN group (*n* = 3). D) Western blotting analysis of PD‐L1 and PTPN2 protein in edited cells. E) Scanned images of 3D tumor spheroids treated by PEI 25K/pDNA, core, PUN, and PUN+MMP‐2 every 20 µm. F) Semi‐quantitative analysis of YOYO‐1 labeled plasmid's fluorescent intensity at different depth. G) Fluorescence signal distribution of TOTO‐3‐plasmids loaded PUN in vivo after intravenous administration. H) The fluorescence images of excised tumor and major organs at 3, 6, 12, 24, and 36 h (*n* = 3) (*P < 0.05, **P < 0.01, ***P < 0.001).

As efficient pDNA expression is a prerequisite for the realization of gene editing, the transfection efficiency of PUN was further determined. To facilitate the assay, plasmid encoding enhanced green fluorescent protein (pEGFP) with similar size to CRISPR/Cas9 plasmid (≈10 kB) was used as the reporter gene. The cells after different treatments were observed under fluorescent microscope (**Figure** **3**A) and analyzed by flow cytometry (Figure 3B). The core exhibited higher transfection ability (81.2 ± 3.3%) than PEI 25K/pDNA (55.7 ± 3.4%) and PEI 1.8K/pDNA (10.8 ± 2.6%) (*P* < 0.001), indicating the advantage of ROS responsiveness in improving transfection. It is worth noting that the transfection efficiency of PUN (72.8 ± 1.7%) was superior to that of PEI 25K/pDNA (*P* < 0.001). Moreover, MMP‐2 preincubated PUN induced significantly increased gene expression (80.0 ± 2.2%) (*P* < 0.01), which suggested that MMP‐2 mediated deshielding of PEG layer is important to get a prominent transfection efficiency. Furthermore, we investigated the transfection efficacy of PUN in vivo condition. EGFP expression in the tumor at 48 h after intravenous injection of normal saline (NS), pDNA, HRMP, and PUN was performed on frozen section. As shown in Figure 3C, PUN group displayed the strongest fluorescence intensity, which may attribute to the multistage responsive functionality of PUN. The above results together testified the preferable transfection efficiency of PUN in vitro and in vivo.

Based on the good transfection ability of PUN, the expression level of PD‐L1 and PTPN2 proteins in genome edited cells were detected by western blot. CRISPR/Cas‐PT was executed in the preparation of core and PUN. After transfection, PUN resulted in lower protein expression of PD‐L1 and PTPN2 than blank samples. Significantly, MMP‐2 pretreated PUN mediated the highest efficacy of the PD‐L1 and PTPN‐2 downregulation, which was more effective than PUN without MMP‐2 (Figure 3D). It suggested that PUN could efficiently downregulate the protein expression of PD‐L1 and PTPN2 in tumor cells owing to the favorable effect of MMP‐2 responsiveness.

Considering the feasibility of PUN in vivo application, a 3D tumor model was performed to explore the penetrating ability of PUN. As shown in Figure 3E, PUN presented a relatively deep penetration, and PUN with MMP‐2 digestion exhibited enhanced tumor‐penetration ability, attributed to the fact that MMP‐2 induced PEG detachment thus re‐exposed RGD facilitated the deep penetration. Additionally, semi‐quantitative analysis of the fluorescence intensity further verified the strongest penetrating ability of MMP‐2‐treated PUN (Figure 3F). Furthermore, the potential of PUN to serve as a targeting delivery system in vivo was evaluated using a subcutaneous xenografted tumor model. With increasing time, PUN progressively accumulated in the tumor site, the highest fluorescence signal appeared around 6 h and was still detained after 24 h (Figure 3G). A reasonable explanation is that upon PUN arriving at the tumor via enhanced permeability and retention (EPR) effect, overexpressed MMPs in tumor would trigger the exposure of RGD to increase the tumor recognition and accumulation. At each time point, the mice were sacrificed to measure ex vivo fluorescence of tumors and main organs. Figure 3H displayed that the highest fluorescence intensity was observed in tumors, and the tumor accumulation lasted until 36 h. The result suggested that PUN was capable of prolonged blood circulation and precise tumor‐targeting ability.

**Figure 4:**
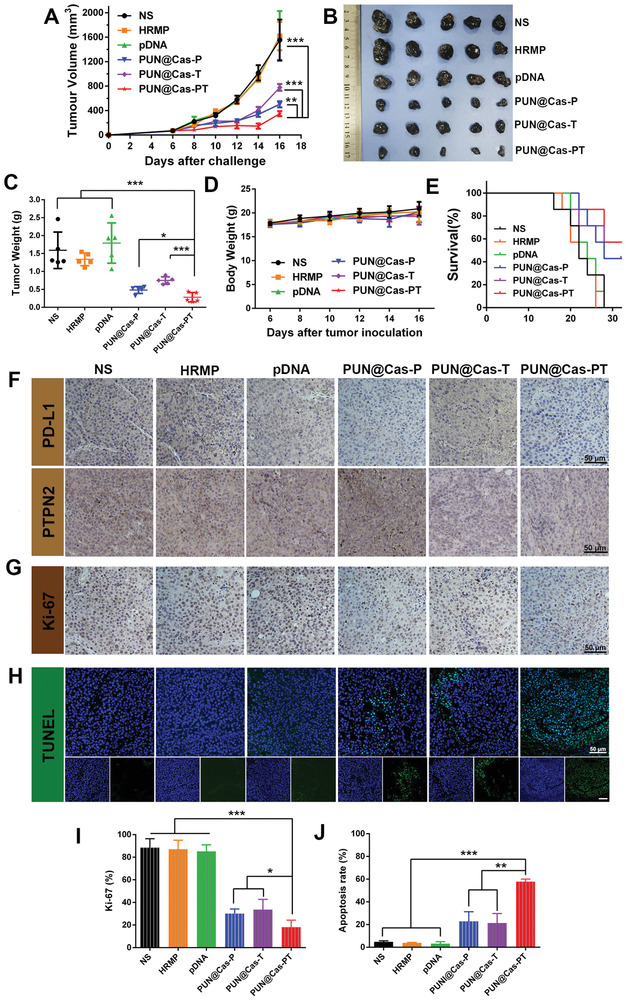
In vivo antitumor efficiency of PUN. A) Tumor volume variation of mice with different treatments including NS, HRMP, pDNA, PUN@Cas‐P, PUN@Cas‐T, and PUN@Cas‐PT intravenous injection (*n* = 5). B) The photograph and C) weight of isolated tumors after the last administration. D) The variety of body weight of mice during the treatment. E) The survival rate of B16‐F10 tumor‐bearing mice after administration (*n* = 7). Immunohistochemical analysis of the tumor slices stained with F) PD‐L1, PTPN2 and G) Ki‐67. H) Representative images of tumor sections stained with TUNEL. I) Percentage of Ki‐67‐positive and J) TUNEL‐positive cells in histological sections of different treatment groups (*P < 0.05, **P < 0.01, ***P < 0.001).

Encouraged by the in vitro editing efficacy and in vivo tumor‐targeting capacity, in vivo antitumor effects were determined on B16‐F10 xenograft tumor model. As provided in **Figure** **4**A, tumors grew wildly in NS‐treated group (1554.2 ± 332.5 mm^3^) and the groups treated with HRMP (1617.8 ± 231.7 mm^3^) and pDNA (1620.2 ± 407.6 mm^3^). In contrast, PUN@Cas‐PT showed the strongest suppression of tumor growth (349.2 ± 41.5 mm^3^) (*P* < 0.001) among all treatments, which was better than PUN@Cas‐P group (502.8 ± 50.0 mm^3^, *P* < 0.01) and PUN@Cas‐T group (778.3 ± 55.5 mm^3^, *P* < 0.001), thus demonstrating its superior antitumor ability. The relatively good inhibition of tumor growth might be ascribed to the cascade amplified immunotherapeutic effect of PUN@Cas‐PT. The photograph of isolated tumors and tumor weight further supported this result (Figure 4B,C). Furthermore, PUN@Cas‐PT treatment did not give rise to any abnormal changes in body weight (Figure 4D), hematoxylin and eosin (H&E) staining sections of main organs and serum biochemistry (Figures S8 and S9, Supporting Information), demonstrating the low toxicity of PUN@Cas‐PT. Additionally, the mice in the control groups showed a short life span of about 28 d, whereas the survival rate of PUN@Cas‐PT group was about 60% (Figure 4E). To further elucidate the enhanced therapeutic efficacy of PUN@Cas‐PT, tumors were subjected to immunohistochemistry (IHC) and immunofluorescence (IF) staining. As presented in Figure 4F, PUN@Cas‐PT group exhibited the lowest expression of PD‐L1 and PTPN2, which confirmed its optimal disruption of PD‐L1 and PTPN2. Importantly, such specific downregulation of PD‐L1 and PTPN2 at the tumor may be ascribed to good tumor targeting performance of PUN@Cas‐PT, multistage responsive release of CRISPR/Cas‐PT as well as the high specificity of sgRNA, which also confirmed the safety of PUN@Cas‐PT in cancer gene therapy. What's more, PUN@Cas‐PT induced the most effective antiproliferation (18.1 ± 6.2%, *P* < 0.001) and pro‐apoptosis effect (57.8 ± 2.2%, *P* < 0.001) (Figure 4G–J). H&E analysis (Figure S10, Supporting Information) further supported the superiority of PUN@Cas‐PT treatment.

**Figure 5:**
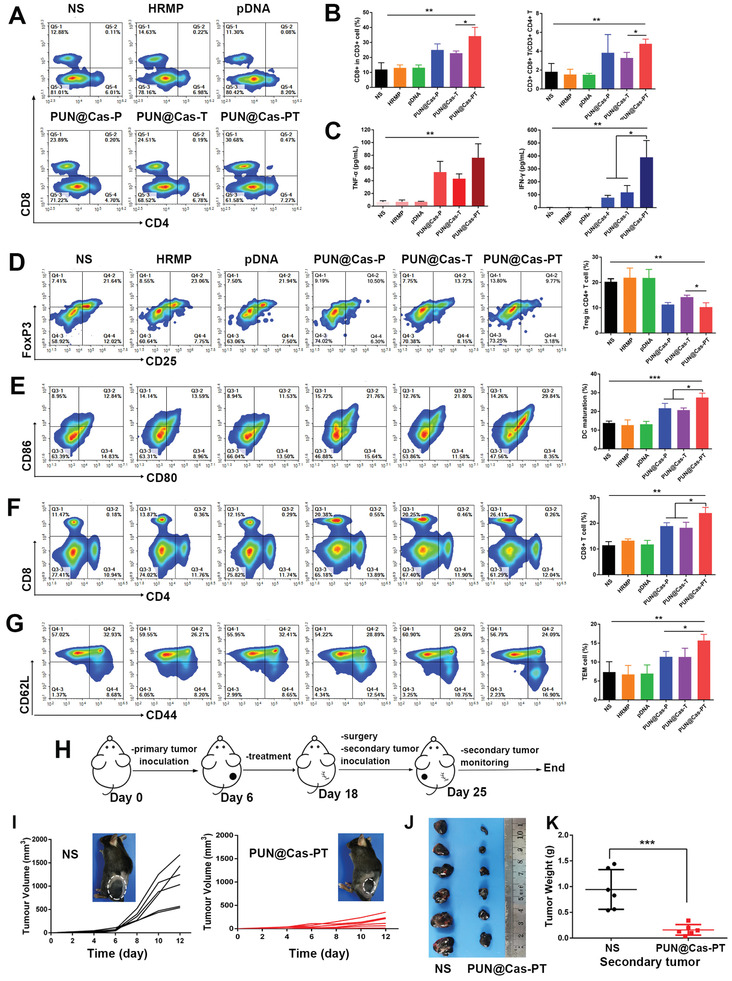
Evaluation of the immune effects of PUN in vivo. Mice were treated by different formulations (NS, HRMP, pDNA, PUN@Cas‐P, PUN@Cas‐T, and PUN@Cas‐PT) (*n* = 5). A) The representative flow cytometric plots of CD8^+^ T cells (CD3^+^CD8^+^) in tumors after treatments. B) The quantification of CD3^+^CD8^+^ T cells and CD3^+^CD8^+^ T/ CD3^+^CD4^+^ T. C) Expression level of TNF‐*α* and IFN‐*γ* in the serum analyzed by ELISA assay. D) Tregs (CD4^+^CD25^+^Foxp3^+^) in tumors detected by flow cytometry and quantitative analysis. E) The mature DCs (CD11C^+^CD80^+^CD86^+^) subpopulation in LNs. F) The flow cytometric analysis of CD8^+^ T cells and G) TEMs (CD3^+^CD8^+^CD44^+^CD62L^−^) in spleens from various groups. H) Schematic illustration of the experimental design for the re‐challenge study (*n* = 6). I) Growth curves of the secondary tumors of the mice treated by NS and PUN@Cas‐PT. The inset images of B16‐F10 xenografted mice recorded at the day 12 post rechallenged. J) The image and K) average weight of the secondary tumors in control and treated group (*P < 0.05, **P < 0.01, ***P < 0.001).

It has been reported that negative regulation of PD‐L1 or PTPN2 is associated with the elicitation of immune response.^[^ ^11^, ^19^ ^]^ To elucidate the adaptive immunity activation mediated by PUN@Cas‐PT, the tumors, spleens, and lymph nodes (LNs) of different groups were collected and analyzed by flow cytometry after one‐week treatment. The percentage of infiltrated cytotoxic T lymphocytes (CD8^+^ T cells, CD3^+^CD4^−^CD8^+^) in PUN@Cas‐PT group (34.2 ± 5.8%) performed a significant increase in contrast to the control groups (NS, 11.9 ± 4.5%; HRMP, 13.0 ± 2.0%; pDNA 13.1 ± 1.9%) (*P* < 0.01) (**Figure** **5**A,B). It was noteworthy that PUN@Cas‐PT group outperformed those groups receiving PUN@Cas‐P (24.9 ± 4.1%) and PUN@Cas‐T (22.8 ± 1.5%) (*P* < 0.05). The ratio of CD8^+^ T/CD4^+^ T in PUN@Cas‐PT was 2.6‐fold higher than that of NS group (*P* < 0.01). Besides, the concentrations of tumor necrosis factor‐*α* (TNF‐*α*) and interferon‐gamma (IFN‐*γ*) in the sera significantly increased when treated by PUN@Cas‐PT than that in NS group (Figure 5C) (*P* < 0.01), and the upregulated secretion of TNF‐*α* and IFN‐*γ* could in turn amplify the adaptive antitumor immunity. PUN@Cas‐PT not only significantly enhanced T cell mediated immune responses but also reversed the tumor‐induced immunosuppression as evidenced by the lowest regulatory T cells (Tregs, CD4^+^CD25^+^Foxp3^+^) (10.2 ± 1.7%) compared to NS (20.3 ± 1.2%) (*P* < 0.01) or PUN@Cas‐T (14.2 ± 0.8%) (*P* < 0.05) (Figure 5D).

Obviously, PUN@Cas‐PT greatly accelerated the dendritic cells (DCs, CD11C^+^CD80^+^CD86^+^) mutation to 27.5 ± 2.3%, which showed superior efficacies to PUN@Cas‐P (21.6 ± 2.7%) (*P* < 0.05) or PUN@Cas‐T (20.7 ± 1.2%) (*P* < 0.05) (Figure 5E). More CD8^+^ T cells in spleens were detected in PUN@Cas‐PT group (24.0 ± 2.2%) compared to that in NS group (11.4 ± 1.4%) (*P* < 0.01), suggested that PUN@Cas‐PT treatment could stimulate effective systemic immune response (Figure 5F). Moreover, a notable increase of effector memory T cells (TEMs, CD3^+^CD8^+^CD44^+^CD62L^−^) in spleen was determined in PUN@Cas‐PT group (15.7 ± 1.6%) than that in NS (7.4 ± 2.7%) (*P* < 0.01), PUN@Cas‐P (11.4 ± 1.4%) (*P* < 0.05), and PUN@Cas‐T group (11.3 ± 2.3%) (*P* < 0.05), implying the induction of immune memory effect (Figure 5G). One explanation for the best efficacy of PUN@Cas‐PT was that, on the one hand, PD‐L1 knockdown in tumor cells attenuated the PD‐1/PD‐L1 interaction, which effectively relieved immune evasion and reinvigorate CD8^+^ T cells to trigger antitumor immune response. On the other hand, PTPN2 downregulation restored the JAK/STAT pathway, thus enhancing the susceptibility of tumor cells to cytotoxic CD8^+^ T cells depending on the sensing of IFN‐*γ*.

To further determine immune memory effects induced by PUN@Cas‐PT, a B16‐F10 tumor‐bearing mouse model was established and subjected to same antitumor therapy as above. On the seventh day after the resection of primary tumors by surgery, B16‐F10 cells were rechallenged into the left flank of the mice in NS and PUN@Cas‐PT group (Figure 5H). Further monitoring of the secondary tumor growth suggested that the progression of tumor in PUN@Cas‐PT group was strikingly suppressed (167.4 ± 125.6 mm^3^) (*P* < 0.001), while the reinoculated tumors in NS group (1084.5 ± 460.8 mm^3^) continued growing rapidly (Figure 5I). The image and weight data of the secondary tumor were obtained before the mice became moribund, which helped to elucidate the immune memory effect against tumor recurrence (Figure 5J,K). The result further demonstrated that PUN@Cas‐PT could activate cascade amplified adaptive immunity and induce long‐term immune memory effect.

## 3 Conclusion

In summary, we have engineered a new type of programmable unlocking nano‐matryoshka‐like system PUN to unleash cascade amplified adaptive antitumor immunity by precisely reversing immunosuppression for permanent, complete, and highly responsive immunotherapy. The CRISPR/Cas9 targeting PD‐L1 and PTPN2 was only activated in tumor tissues benefiting from the programmable unlocking properties of PUN in response to internal stimuli and external triggers. Like a successive unlocking way, the stable PUN in long circulation underwent exfoliation of stealthy layer under the action of MMPs to expose target motif, thus facilitating tumor‐specific accumulation and deep penetration and internalization. Upregulated HAase triggered the second‐stage unlocking response to realize charge conversion for breaking intracellular barriers (i.e., lyso/endosomes). The intracellular ROS stimulated the final breakup and concurrent release of CRISPR/Cas9 for effective gene editing. These characteristic performances significantly improved the accumulation of PUN@Cas‐PT to enhance the gene‐editing efficiency at tumor site as well as attenuated off‐targeted effects by reducing the undesired activations of CRISPR/Cas‐PT in nontargeted organs (e.g., heart, liver, spleen, lung, and kidney). The intervention of PD‐1/PD‐L1 pathway induced by PD‐L1 knockdown as well as the sensitization effect on IFN‐*γ* mediated by deletion of PTPN2 occurred simultaneously in the tumor, which could cascade amplify the adaptive antitumor immunity. All in all, PUN exhibited superior in vitro and in vivo efficacy against melanoma, which provided a promising paradigm for permanent, complete, and highly responsive multitargeted ICB immunotherapy.

## Conflict of Interest

The authors declare no conflict of interest.

4

## Supporting information

Supporting Information

## Data Availability

Research data are not shared.
