# Supplementary material for: Programmable Unlocking Nano‐Matryoshka‐CRISPR Precisely Reverses Immunosuppression to Unleash Cascade Amplified Adaptive Immune Response
Source: Adv Sci (Weinh). 2021 May 14;8(13):2100292. doi: 10.1002/advs.202100292 (PMC8261501; doi:10.1002/advs.202100292)
Supplement: Supplementary file 1 — Supporting Information [file ADVS-8-2100292-s001.pdf]

© 2021 Wiley-VCH GmbH

ADVANCED SCIENCE
Open Access

Supporting Information

for *Adv. Sci.,* DOI: 10.1002/advs.202100292

Programmable Unlocking Nano-matryoshka-CRISPR Precisely Reverses Immunosuppression to Unleash Cascade Amplified Adaptive Immune Response

*Jin Yang, Zhike Li, Meiling Shen, Yan Wang, Li Wang, Jiamiao Li, Wen Yang, Jie Li, Haijun Li, Xinxin Wang, Qinjie Wu, Changyang Gong**

Supporting Information

Programmable Unlocking Nano-matryoshka-CRISPR Precisely Reverses Immunosuppression to Unleash Cascade Amplified Adaptive Immune Response

Jin Yang, Zhike Li, Meiling Shen, Yan Wang, Li Wang, Jiamiao Li, Wen Yang, Jie Li, Haijun Li, Xinxin Wang, Qinjie Wu, Changyang Gong *

State Key Laboratory of Biotherapy and Cancer Center, West China Hospital, Sichuan University, and Collaborative Innovation Center for Biotherapy, Chengdu, 610041, P. R. China

* To whom correspondence should be addressed (C Gong). E-mail: chygong14@163.com.

Experimental Section

Materials. bPEI (MW 1.8kDa and 25kDa) was purchased from Alfa Aesar Co., Inc (Ward Hill, MA, USA). HA (MW 35 kDa) was obtained from Shandong Freda Biochem Co., Ltd (Shandong, China). MPEG-mal (Mn = 2000) was provided by Ponsure Biotech, Inc (Shanghai, China). The cleavable RGD-MMP peptide (CGPLGVRGRGDK) was synthesized by Chinapeptides Co. Ltd (Shanghai, China). Human MMP-2 was bought from PeproTech (USA). YOYO-1, TOTO-3 and 4, 6-diamidino-2-phenylindole dihydrochloride (DAPI) were purchased from Invitrogen (USA). A ROS Assay Kit was purchased from was ordered from Biovision (USA). All antibodies for flow cytometry analysis and enzyme-linked immunosorption assay (ELISA) kits for cytokines were purchased from biolegend (USA). All other chemicals were of analytical grade and used without purification.

Cell Culture and Animals. B16-F10 cells (obtained from American Type Culture Collection, Rockville, MD) were cultivated in Dulbecco's Modified Eagle Medium (DMEM) supplemented with penicillin-streptomycin and 10% fetal bovine serum (FBS, Gibco, USA). The cells were kept under a humidified atmosphere containing 5% CO_2 at 37℃.

BALB/c nude mice and C57BL/6 mice (female, 6-8 weeks old) were purchased from HFK Bioscience Co., Ltd. (Beijing, China) and fed in specific pathogen free condition All animal experiments performed following the protocols approved by the Institutional Animal Care and Treatment Committee of Sichuan University (Chengdu, P. R. China).

Engineering of CRISPR/Cas9 system. To select the optimal sgRNA for efficient genome editing, three pairs of oligonucleotide sequences were designed using the website tool (http://crispr.mit.edu) based on the analysis of genomic sequence of PD-L1 and PTPN2, respectively. The sequences were ligated into the BbsI restriction site of pX333 (Addgene, plasmid

#64073) for generating the sgRNA expressing vector (CRISPR/Cas-P and CRISPR/Cas-T). The sgRNA2 (5'-CACCGAGTACACCACTAACGCAAGC-3') targeting PD-L1 and sgRNA1 (5'-CACCGCCATGACTATCCTCATAGAG-3') targeting PTPN2 with satisfactory efficiency were selected as the optimal candidates for constructing the multi-editing CRISPR/Cas9 system. In brief, the two selected sgRNAs were annealed and embedded into the BbsI/ Eco31I -predigest PX333 vector in order to get CRISPR/Cas-PT. Similarly, direct sequencing and T7EI analysis were conducted after acquiring the amplified PCR products.

Synthesis of PR. Firstly, thioketal linker was synthesized referring to previous work.[1] The carboxyl groups of the thioketal linker (50mg, 0.198mmol) were then activated by DCC/NHS and further reacted with the amino groups of PEI 1.8K (712.8mg, 0.396mmol) in anhydrous DMSO (10 mL) for 48 h to obtain PEI-based ROS-cleavable polymer PR. Followed by the filtration and purification, the product was lyophilized and characterized by ^1H NMR spectroscopy and FT-IR.

Synthesis of HRMP. The synthesis of MMPs-activated targeted polymer HRMP required two-step reactions. Briefly, HA (50mg, 0.124mmol) was first activated by EDCI/NHS system for 2 h at room temperature (RT). After that the dissolved RGD-MMP peptide (37.6mg, 0.031mmol) was added dropwise and stirred overnight. The reaction mixture was then purified by dialysis and the product was obtained by lyophilization. HRMP were then synthesized by a conjugation between the thiol group of the resulting product HA-RGD-MMP and the maleimide group of MPEG-mal (62mg, 0.031mmol). The reaction was carried out at RT for 24 h, followed by dialyzed in distilled water and freeze-drying to obtain the white powder. The final product was confirmed by ^1H NMR and FT-IR.

Preparation of PUN. For preparation of the core at first, 50 μL of the cationic polymer PR solution (80 μg mL^{-1}) was gently mixed with equal volume of pDNA solution (20 μg mL^{-1}) and

incubated for another 25 min at RT. To form the stable PUN, 100 μL of the HRMP solution (400 μg mL^{-1}) was next added into the freshly prepared core and incubated for 25 min at RT. The particle size and zeta potential of the nanoparticles were detected by DLS measurements ((Malvern, Zetasizer NanoZS ZEN 3600, U.K.). Moreover, the morphologies were observed using TEM (JEOL JEM-100CX, Japan).

Programming unlocking responsiveness of PUN. The multistage responsive ability of the PUN was investigated by monitoring the changes in particle size, zeta potential and morphology under different conditions. The prepared PUN was separately incubated with MMP-2 and HAase according the manufacturer's instruction. After the incubation, the hydrodynamic diameter and surface charge of the PUN were evaluated by DLS. The TEM images were token subsequently. To verify the ROS sensitivity, the core was incubated in 25 mM H_2O_2 solution containing 1.6 μM $CuCl_2$. The particle shape was recorded by TEM after incubating for 12 h at 37°C.

Cytotoxicity analysis. B16-F10 cells were seeded in 96-well plates at a density of 5000 cells per well and allowed to grow overnight. The original medium was then replaced by the fresh DMEM medium containing different polymers at varied concentrations (PEI 25K, PEI 1.8K and PR: 0-20 μg mL^{-1}; HRMP: 0-400 μg mL^{-1}), followed by incubating for further 24 h. MTT solution (5 mg mL^{-1}) was added into each plate and incubated for 2 h. The quantification of the cell viability was obtained by measuring the absorbance at 570 nm on a Multiskan MK3 microplate absorbance reader (Thermo Scientific, USA).

Hemolysis Assays. The freshly prepared PUN was added into 2% rabbit erythrocyte suspension to achieve different concentrations (25, 50, 100, 200, 400 and 800 μg mL^{-1}). 0.9% NaCl solution and distilled water were used as negative and positive control, respectively. After 2 h incubation at 37°C, the samples were centrifuged in tube. The photograph was token immediately and the

supernatant of each sample was measured on a UV/Vis spectrophotometer (SHIMADZU, Japan) at absorbance 545 nm.

Analyses of cellular uptake and endosomal escape. The cellular uptake was evaluated by flow cytometry. pDNA was labeled with YOYO-1 following the instruction. Different nanoparticles were prepared according to the method above and then added into the cells with or without MMP-2 pre-treating. After incubation for 1 h in dark, the cells were collected and analyzed using a flow cytometer (NovoCyte™, ACEA Biosciences, USA).

For endosome escape capability analysis, B16-F10 cells were seed into 35 mm confocal dishes (Φ =15 mm) and cultured for 24 h before the experiment. The core and PUN loaded with YOYO-1 labeled plasmids were incubated with cells. Thereafter, LysoTracker Red and DAPI were used for staining the lyso/endosomes and nuclei at designed time points, respectively. The cells were rinsed and fixed with 4% paraformaldehyde for 15 min and then observed under CLSM (Olympus, Japan).

***In vitro* and *in vivo* gene transfection.** To investigate the transfection efficiency of the prepared nanoparticles under different conditions (with or without MMP-2), B16-F10 cells were seeded into 12-well plates prior to transfection. The nanoparticles containing same amount of pEGFP were then added into cells and cultured in FBS-free medium for 6 h. Hereafter, the medium was replaced with fresh DMEM medium and cultured for another 24 h. The EGFP expression of cells were observed under the fluorescence microscope (Olympus, Japan). The cells were collected and analyzed by flow cytometry.

To determine the gene transfection *in vivo*, different formulations were intravenously injected into B16-F10 xenograft mice. At 48 h later, the tumors were harvested and embedded in tissue freezing medium immediately. The fluorescence imaging was performed on frozen tumor sections.

Western-blot assay. To evaluate the expression of PD-L1 and PTPN2 proteins, western-blot

analysis was performed after the transfection with different nanoparticles (core and PUN) as previously described. Total protein extracts were separated and transferred to poly (vinylidene fluoride) (PVDF) membranes (Millipore, USA). Membranes were incubated with corresponding antibody following the well-established protocol. Blots were visualized by enhanced chemiluminescence (PerkinElmer, USA). The expression of β-actin in the cells was also measured as a control.

***In vitro* tumor penetration.** B16-F10 cells were cultured to form stable 3D spheroids according the method reported.[2] Then, PEI 25K/pDNA, core, PUN (with or without MMP-2 pre-treating) were added into the FBS-free medium and incubated with the selected spheroids for 6 h. The tumor spheroids were then washed by phosphate-buffered saline (PBS), fixed with 4% paraformaldehyde, and put onto slide glasses for further observation under CLSM. The images were acquired using Z-stack automatic scanning system and analyzed by ImageJ for semi-quantitative analysis.

Biodistribution of PUN. To determine the tumor accumulating capability of PUN, an A375 xenograft athymic nude mouse model was established and the plasmids were labeled with TOTO-3 for visualizing the distribution of PUN *in vivo*. Thereafter, mice were grouped and intravenously injected with the formulation. After anesthetized by isoflurane, images of the mice were captured with IVIS Lumina imaging system (PerkinElmer, USA) at the time of 3, 6, 12, 24 and 36 h post injection, respectively. Meanwhile, the fluorescence intensity of major orangs (hearts, livers, spleens, lungs, and kidneys) *ex vivo* was observed with the imaging instrument at each time point.

***In vivo* anti-tumor immunotherapy.** The antitumor activity of the formulations was investigated on a subcutaneous B16-F10 melanoma cancer model. Experiment began after inoculation for 6 days. The mice were randomly distributed into 6 groups for treatment with various formulations: NS, HRMP, pDNA, PUN@Cas-P, PUN@Cas-T and PUN@Cas-PT (plasmid dose = 5

μg per mouse). The administration of the above formulations was conducted every two days by tail-vein injection. Tumors volume (*V*) was calculated to be $V = 0.5 \times \text{major axis} \times \text{minor axis}^2$ and the body weights were recorded at the same time. The mice were euthanatized before reaching the moribund state. The tumors were collected followed immediately photographed and weighed. In addition, tumors and major organs were fixed and sectioned into slices for H&E, IHC and terminal deoxynucleotidyl transferase-mediated deoxyuridine triphosphate nick-end labeling (TUNEL) staining according to the protocols provided by manufacture. Moreover, to determine the survival rates, the tumor-bearing mice received the same treatment as above and were monitored every day. The dates of mortality were recorded until the end of the experiment.

Immune response analysis *in vivo*. For immune response analysis *in vivo*, the B16-F10 tumor-bearing mice were randomly grouped and intravenously injected with the formulations as the abovementioned doses. Different treatments were carried out on day 6, day 9 and day 11 after the tumor cells inoculation. Tumors, spleens and LNs of different groups were harvested, grinded and filtered to obtain single-cell suspension. Treated with Red Blood Cell Lysis Buffer (Beyotime), the cells were then washed with PBS and stained with corresponding antibodies following the standard protocol. Following staining, cells were washed again and analyzed using flow cytometer. Additionally, TNF-α and IFN-γ content in the serum of each group was detected using the corresponding ELISA Kits.

Immune memory effect. To further assess the immune memory effect, the tumor-bearing mice were established and divided into two groups including NS and PUN@Cas-PT. The mice were treated as the same procedure applied for anti-tumor immunotherapy *in vivo*. Subsequently, the primary tumors were removed by surgery on the day after the last administration. Seven days after the operation, B16-F10 cell suspension was rechallenged into the left side of each mouse. The

volumes variation of the second tumors in each mouse were recorded every other day. The monitoring stopped at the endpoint when the mice became moribund and the tumors were isolated from the mice for imaging and weighing.

Statistical analysis. The statistical analysis was carried out using GraphPad Prism software (San Diego, CA) and data were expressed as the mean ± SD. Difference were assessed using one-way analysis of variance (ANOVA) and the two-tailed student's t-test. Asterisks (*) denote the statistical significance (* , ** and*** are indicated P <0.05, P <0.01 and P <0.001).

References

[1] X. Ling, S. Zhang, P. Shao, P. Wang, X. Ma, M. Bai, Tetrahedron letters 2015, 56, 5242.

[2] L. Li, X. Li, Y. Wu, L. Song, X. Yang, T. He, N. Wang, S. Yang, Y. Zeng, Q. Wu, Z. Qian, Y. Wei, C. Gong, Theranostics 2017, 7, 1633.

Supporting Figures

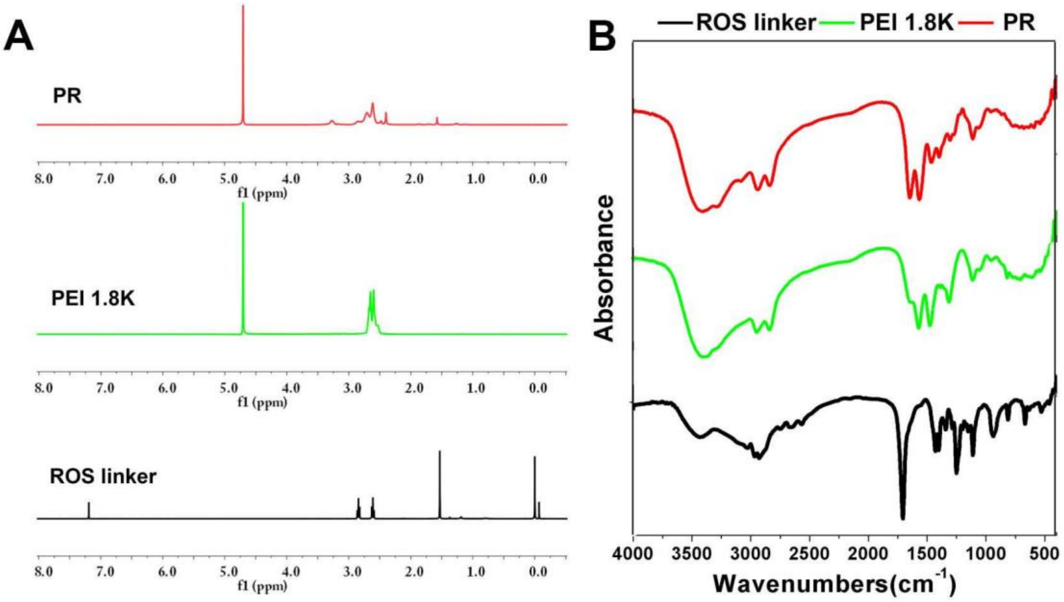

Figure S1 Physicochemical characterization of PR. A) ^1H NMR spectra (300 MHz, $CDCl_3$) of ROS linker and ^1H NMR (300 MHz, D_2O) of PEI 1.8K and PR. B) FT-IR of ROS linker, PEI 1.8K and PR.

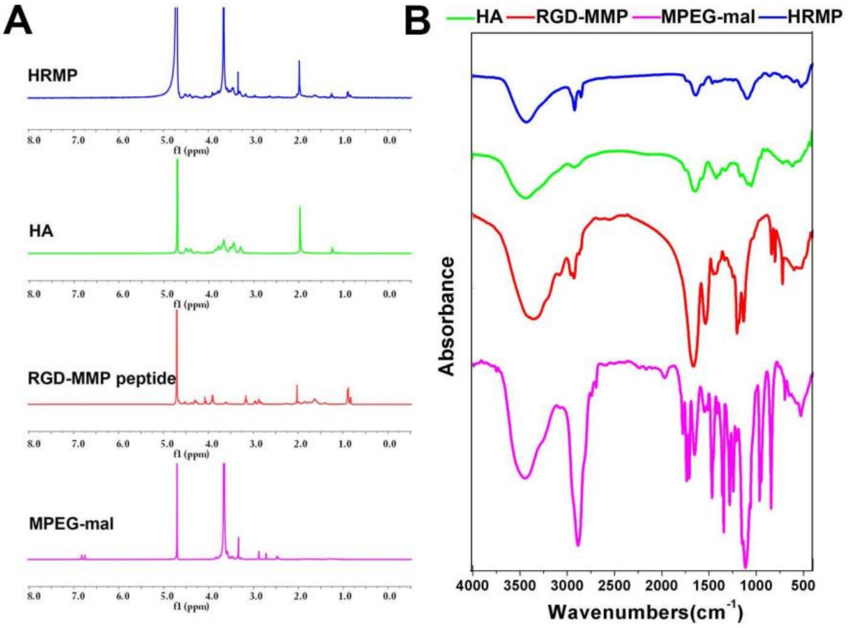

Figure S2 Characterization of HRMP. A) ^1H NMR spectra (300 MHz, D_2O) and B) FT-IR of HA, RGD-MMP peptide, MPEG-mal and HRMP.

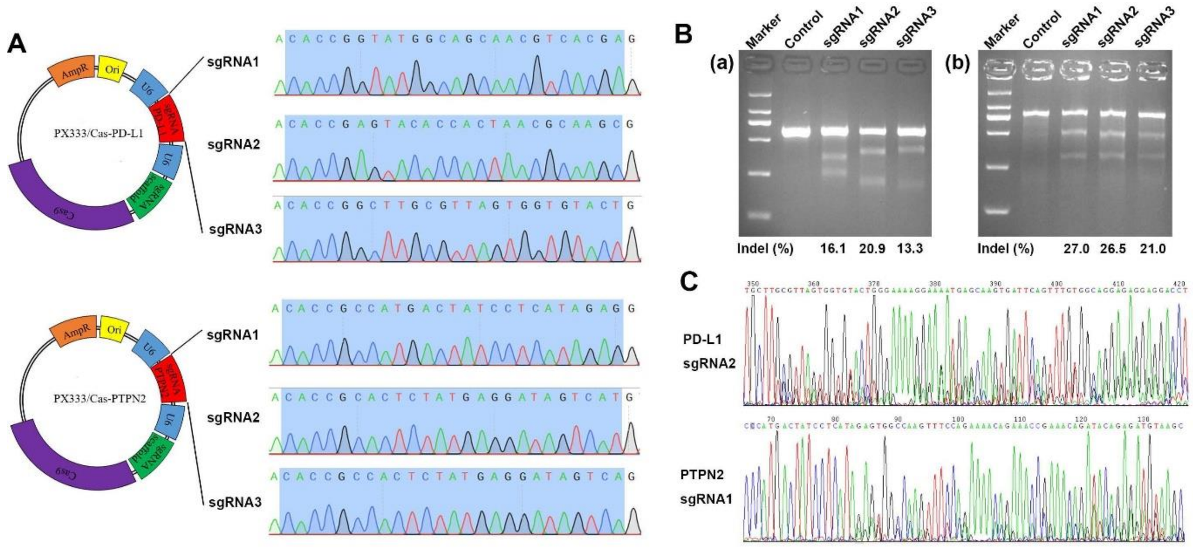

Figure S3 Construction of CRIPSR/Cas9 plasmid targeting PD-L1 and PTPN2. A) Sequencing results of constructed plasmids with different sgRNAs. B) T7EI cleavage assay of disruption rate of sgRNAs in a) CRISPR/Cas-P and b) CRISPR/Cas-T, respectively. C) Sanger sequencing result of the PCR amplicon after transfected with optimal plasmid CRISPR/Cas-P and CRISPR/Cas-T.

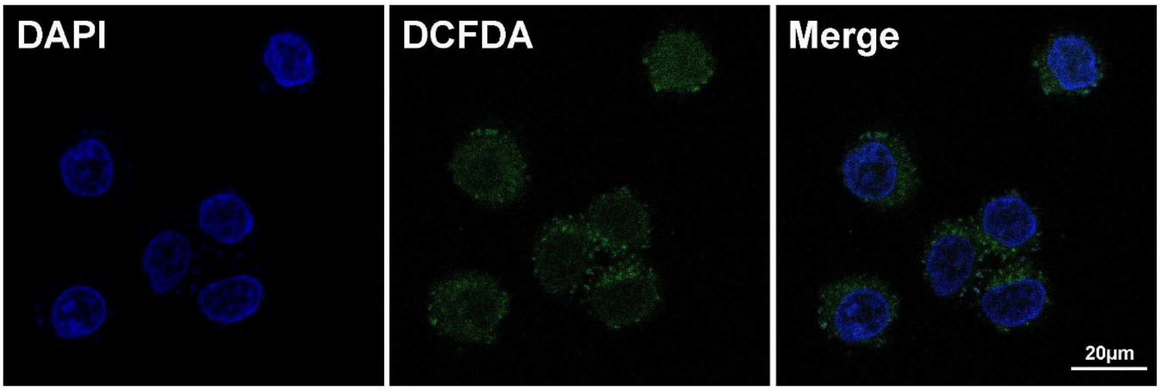

Figure S4 Detection of endogenous ROS. Confocal microscope images of B16-F10 cells after incubation with DCFDA as an ROS probe. The nuclei were stained with DAPI (blue).

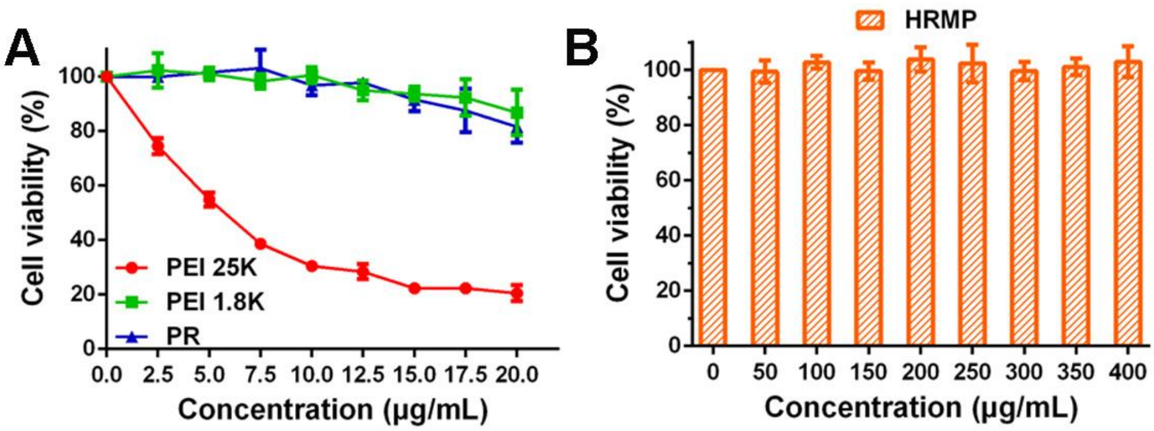

Figure S5 MTT assay of cytotoxicity. A) PEI 25K, PEI 1.8K, PR and B) HRMP against HEK-293 cells.

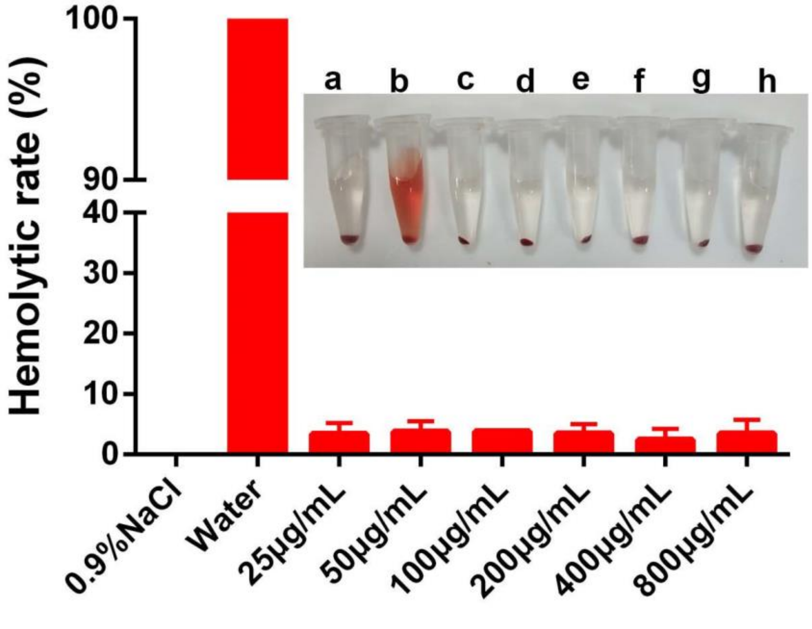

Figure S6 Hemolysis assay. The hemolytic rate of the 2% rabbit red cell suspension after incubated with different concentrations of PUN. The insert image presented the phenomenon after centrifugation ("a-f" referred to the sample containing water, 0.9% NaCl and different concentrations of PUN).

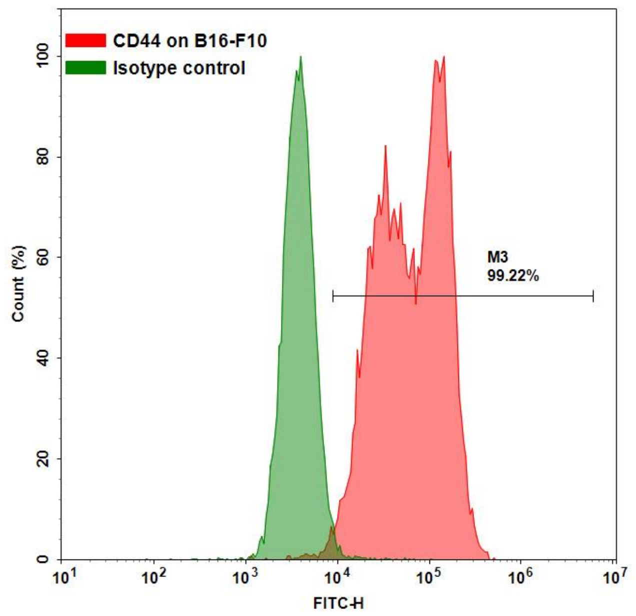

Figure S7 Evaluation of the expression level of CD44 receptor on B16-F10 cells by flow cytometry. Isotype control is shown in green.

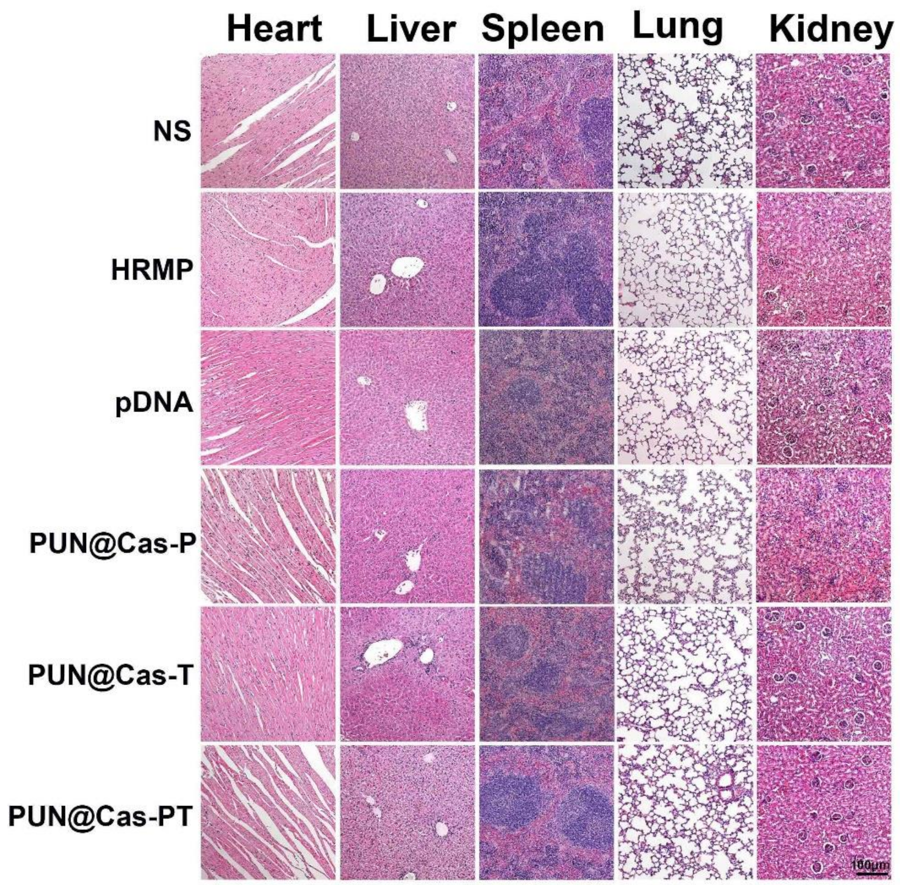

Figure S8 H&E staining images of major organs.

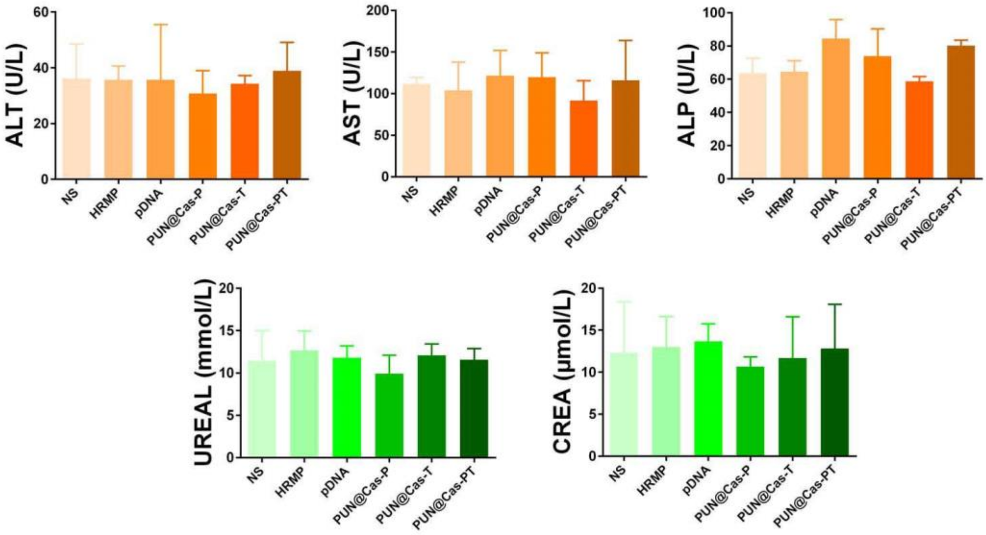

Figure S9 Serum biochemistry analysis after different treatments.

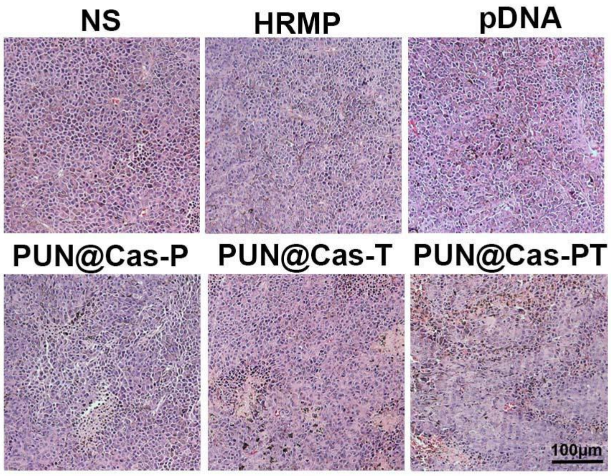

Figure S10 Representative images of H&E-stained tumor tissues of tumor-bearing mice after various treatments.
